# Noninvasive assessment of classic and high PPROM using cervicovaginal podocalyxin and nephrin: Findings from a prospective observational study

**DOI:** 10.1097/MD.0000000000045383

**Published:** 2025-10-24

**Authors:** Zercan Kali, Pervin Karli, Ümran Karabulut, Fatma Tanilir Çağiran, Pinar Kirici, Serhat Ege

**Affiliations:** a Department of Obstetrics and Gynecology, Private Gözde Hospital, Malatya, Turkey; b Private Obstetrics and Gynecology Clinic, Samsun, Turkey; c Department of Obstetrics and Gynecology, Private Acibadem Hospital, Kayseri, Turkey; d Private Obstetrics and Gynecology Clinic, Diyarbakir, Turkey; e Department of Obstetrics and Gynecology, Turgut Özal University, Malatya, Turkey; f Department of Gynecology, Dicle University, Diyarbakir, Turkey.

**Keywords:** bronchopulmonary dysplasia, fetal membranes, nephrin, podocalyxin, premature, respiratory distress syndrome, ROC curve, rupture

## Abstract

This study assesses the diagnostic and prognostic value of cervicovaginal amniotic fluid (CVAF) podocalyxin (PDX) and nephrin levels in pregnancies with classic and high preterm premature rupture of membranes (PPROM), focusing on neonatal outcomes. This prospective study included 144 singleton pregnancies between 22 and 34 weeks, classified as classic PPROM (n = 74), high PPROM (n = 32), and controls (n = 38). CVAF and serum samples were analyzed using enzyme-linked immunosorbent assay to quantify PDX and nephrin levels. Receiver operating characteristic curves evaluated diagnostic performance. Logistic regression identified predictors of respiratory distress syndrome and bronchopulmonary dysplasia. CVAF PDX and nephrin levels were significantly higher in the classic PPROM group (35.05 ± 5.55 and 12.88 ± 3.85 ng/mL, respectively) compared to high PPROM and control groups. Receiver operating characteristic analysis demonstrated excellent diagnostic performance for distinguishing classic PPROM, with area under the curve values of 0.92 (95% confidence interval [CI]: 0.88–0.96) for PDX and 0.93 (95% CI: 0.89–0.97) for nephrin. In multivariable logistic regression, elevated PDX was independently associated with bronchopulmonary dysplasia (odds ratio = 1.32, 95% CI: 1.10–1.59), while elevated nephrin predicted respiratory distress syndrome (odds ratio = 1.18, 95% CI: 1.02–1.36). These findings support their utility as noninvasive biomarkers for both diagnosis and risk stratification in PPROM. CVAF PDX and nephrin demonstrated significant diagnostic and prognostic value in differentiating PPROM subtypes and may be useful for neonatal risk stratification. These findings suggest that CVAF PDX and nephrin levels may serve as noninvasive tools for early identification of high-risk PPROM cases, potentially guiding timely intervention and targeted neonatal care.

## 1. Introduction

Preterm premature rupture of membranes (PPROM) is a major obstetric complication, accounting for approximately 25% to 30% of all preterm births and contributing significantly to neonatal morbidity and mortality.^[1,2]^ Traditionally, PPROM is defined as the spontaneous rupture of fetal membranes before 37 weeks of gestation and before the onset of labor.^[3]^ However, recent clinical observations suggest that PPROM is not a uniform condition. In particular, a clinical variant known as “high PPROM” is characterized by confirmed membrane rupture despite preserved amniotic fluid volume on ultrasound and the absence of overt vaginal fluid leakage.^[4–6]^ This atypical presentation often leads to diagnostic uncertainty and may delay appropriate clinical intervention.^[7–9]^ Although not yet standardized in major international guidelines, this subclassification has been increasingly recognized in recent literature and tertiary care practice as a clinically meaningful phenotype, particularly in cases where conventional diagnostic tools are inconclusive^.[3,8]^

Although current diagnostic tools such as IGFBP-1 and PAMG-1 are widely used in clinical practice for the diagnosis of PPROM, their accuracy is limited, particularly in atypical or high cases where fluid leakage is minimal or intermittent. This diagnostic challenge highlights the need for novel, pathophysiology-based biomarkers that can directly reflect the structural integrity of fetal membranes.^[10,11]^

Podocalyxin (PDX) is a CD34-related transmembrane sialomucin glycoprotein involved in epithelial polarity and barrier integrity. It is primarily expressed in renal podocytes, but it has also been detected in the placenta and amniotic membranes. Nephrin is a key structural component of the slit diaphragm within the glomerular filtration barrier, playing a critical role in regulating intercellular cohesion and permeability. The presence of these proteins in fetal tissues suggests that they may also contribute to the maintenance of fetal membrane integrity during pregnancy.^[9,12,13]^

Moreover, membrane rupture is often accompanied by intrauterine inflammation and endothelial dysfunction, which may negatively affect fetal lung development and lead to neonatal complications such as respiratory distress syndrome (RDS) and bronchopulmonary dysplasia (BPD). Therefore, elevated levels of PDX and nephrin in CVF may reflect not only mechanical membrane damage but also underlying biological processes contributing to these complications.^[14,15]^

This study aims to evaluate serum and CVF levels of PDX and nephrin in patients diagnosed with classic and high PPROM, compared to a healthy pregnant control group. By clinically stratifying PPROM into meaningful subtypes, we aim to assess whether these biomarkers can enhance the diagnosis of high PPROM, particularly in cases where conventional diagnostic methods like AmniSure fall short. Ultimately, this research seeks to determine the potential utility of PDX and nephrin as complementary or alternative biomarkers for the early and accurate identification of PPROM subtypes.

## 2. Methods

### 2.1. Research design

This prospective observational study was conducted to evaluate the diagnostic and prognostic value of cervicovaginal amniotic fluid (CVAF) PDX and nephrin levels in pregnancies complicated by classic and high PPROM. The study was carried out between October 2024 and April 2025 at the Department of Obstetrics and Gynecology, Private Gözde Hospital in Malatya, Turkey. The research design was structured to reflect routine clinical practice, without any form of intervention or randomization, thereby allowing the natural progression of the condition to be observed in a real-world setting.

Participants were prospectively enrolled and categorized into 3 groups based on clinical findings and ultrasonographic criteria: classic PPROM, high PPROM, and a healthy control group. This stratification enabled a comparative evaluation of biomarker profiles across varying degrees of fetal membrane compromise. The design allowed for the simultaneous assessment of both diagnostic utility and prognostic potential of the selected biomarkers in a clinically meaningful context.

### 2.2. Population

A total of 162 pregnant women between 22 and 34 gestational weeks were initially assessed for eligibility. Following the application of predefined inclusion and exclusion criteria, 18 participants were excluded due to factors such as vaginal bleeding, fetal or placental anomalies, recent cervical cerclage, extended latency period (>48 hours) between membrane rupture and hospital admission (except in high PPROM), contaminated CVF samples, suspected fetal renal disorders (e.g., congenital nephrotic syndrome), maternal infectious diseases (HIV, hepatitis B), recent sexual activity, intrauterine fetal distress, placenta previa, or signs of imminent labor.

**Figure 1.**
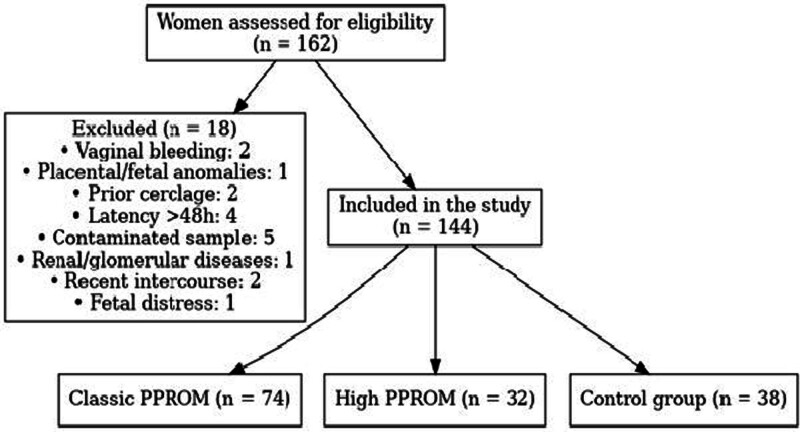
The flowchart below illustrates the selection of participants and their distribution into 3 groups: classic PPROM, high PPROM, and the control group. PPROM = preterm premature rupture of membranes.

After the exclusion of these individuals, 144 eligible participants were enrolled in the study and categorized into 3 groups: classic PPROM (n = 74), high PPROM (n = 32), and healthy controls (n = 38). The complete participant selection process, including reasons for exclusion and final group allocation, is depicted in the study flowchart (Fig. 1). This careful refinement ensured that the study population was both clinically homogeneous and methodologically sound for comparative biomarker analysis.

A flowchart depicting the enrollment process and reasons for exclusion is provided in Figure 1.

Inclusion criteria comprised singleton pregnancies – regardless of conception method (spontaneous or IVF) – with confirmed PPROM or normal pregnancy. Exclusion criteria included:

• Vaginal bleeding.
• Placental or fetal anomalies.
• Prior cervical cerclage.
• Latency period > 48 hours between fluid leakage and admission (except for high PPROM).
• Contaminated (bloody or mucus-filled) CVAF samples.
• Suspected or confirmed fetal renal anomalies or inherited glomerular diseases (e.g., congenital nephrotic syndrome).
• Infectious diseases such as HIV or active hepatitis B.
• Recent sexual activity (<24 hours).
• Intrauterine fetal distress.
• Placenta previa or active vaginal bleeding.
• Unstable vital signs, eclampsia, placental abruption, fetal distress, or imminent labor (cervical dilation > 5 cm).

Gestational age was determined based on the last menstrual period and confirmed by first-trimester ultrasonography where available.

### 2.3. Diagnosis of PPROM and subgroup classification

The diagnosis of PPROM was made via sterile speculum examination to detect pooling of amniotic fluid in the posterior vaginal fornix or leakage through the cervical os.^[3]^ In cases where fluid leakage was not visible, a nitrazine test was performed. Coughing or fundal pressure was applied if necessary to provoke leakage. Manual vaginal examination was avoided to reduce infection risk.

Classic PPROM was defined as fluid leakage with ultrasonographic evidence of oligohydramnios (maximum vertical pocket < 2 cm).

High PPROM was diagnosed in the presence of fluid pooling or a positive nitrazine test but normal amniotic fluid volume.

All PPROM patients received prophylactic antibiotics and antenatal corticosteroids. Delivery was induced upon signs of chorioamnionitis, fetal distress, or prolonged latency. High PPROM patients were monitored similarly due to the risk of progression to classic PPROM.

The control group consisted of healthy singleton pregnancies between 22 and 34 weeks of gestation without any obstetric complications or membrane rupture. In the control group, cervicovaginal secretions were obtained via posterior fornix swabbing during routine antenatal examination. All participants were fully informed about the nature and purpose of the sampling, and written informed consent was obtained prior to the procedure in accordance with institutional ethical guidelines

RDS and BPD were diagnosed by the attending neonatologist based on standard clinical criteria RDS was diagnosed according to the European Consensus Guidelines on the management of RDS.^[16]^ The diagnosis was based on the presence of respiratory distress, the need for supplemental oxygen, and compatible radiographic findings. BPD was defined in accordance with the National Institutes of Health consensus criteria^[17]^ as oxygen dependency at 36 weeks of postmenstrual age.

### 2.4. Sample collection and biomarker analysis

CVAF samples were collected from the posterior vaginal fornix using sterile Dacron swabs (Puritan Medical, Falmouth) placed for 15 seconds, before any digital examination. Samples contaminated with blood, urine, or mucus were excluded. The swabs were placed in cryotubes containing 1 mL of sample buffer and stored at −80°C until analysis.

Maternal venous blood samples were collected concurrently. All samples were centrifuged at 3500 rpm for 5 minutes and stored at −80°C.

Quantitative sandwich enzyme-linked immunosorbent assay kits (Sunred Biotechnology, Shanghai, China) were used to measure PDX and nephrin levels in both CVAF and serum samples:

• PDX assay range: 0.2 to 60 ng/mL; sensitivity: 0.153 ng/mL.
• Nephrin assay range: 0.2 to 40 ng/mL; sensitivity: 0.16 ng/mL.

All assays had intra-assay coefficient of variation < 10% and inter-assay coefficient of variation < 12%. Absorbance was read at 450 nm using a BioTek ELx800 microplate reader (BioTek Instruments, Winooski). Results were expressed in ng/mL.

### 2.5. Statistical analysis

Statistical analyses were performed using IBM SPSS Statistics for Windows, Version 27.0 (IBM Corp., Armonk) and GraphPad Prism, Version 8.0 (GraphPad Software, San Diego). Data distribution was assessed using the Kolmogorov–Smirnov test. Depending on distribution, either the independent samples *t*-test or the Mann–Whitney *U* test was used for group comparisons. Categorical variables were evaluated using the chi-square test or Fisher exact test.

Spearman correlation analysis was used to explore associations between biomarker levels and clinical outcomes. Binary logistic regression was performed to identify independent predictors of neonatal outcomes (e.g., RDS, BPD), adjusting for maternal age, anemia, and hypertensive disorders. Receiver operating characteristic (ROC) curve analysis was conducted to assess the diagnostic accuracy of CVAF PDX and nephrin concentrations. A *P*-value < .05 was considered statistically significant.

## 3. Results

As shown in Table 1A and B, baseline demographic characteristics, including maternal age, gravidity, and parity, were comparable across the 3 study groups (*P* > .05 for all). There were no statistically significant differences in the proportions of primigravida or multiparous participants among the classic PPROM (Group 1), high PPROM (Group 2), and control groups.

**Table 1:** Demographic, clinical, and biochemical characteristics of study groups.

Variable	Group 1: classic PPROM (n = 74)	Group 2: high PPROM (n = 32)	Group 3: control (n = 38)	*P* value
*A. Continuous variables*				
Maternal age (yrs)	29.67 ± 5.93	30.92 ± 2.50	29.7 ± 4.1	.30
Gestational age at birth (weeks)	30 (26–32)	32 (30–34)	38 (37–39)	<.001*
Birth weight (g)	1570 ± 567.6	1892.3 ± 138.2	3035 ± 320	.047*
Maternal serum podocalyxin (ng/mL)	8.6 ± 1.5	8.7 ± 1.4	8.5 ± 1.3	.16
Maternal serum nephrin (ng/mL)	4.2 ± 1.0	4.1 ± 0.9	4.3 ± 0.8	.74
CVAF podocalyxin (ng/mL)	35.05 ± 5.55	23.42 ± 3.77	10.57 ± 3.21	.002*
CVAF nephrin (ng/mL)	12.88 ± 3.85	7.58 ± 2.91	5.84 ± 2.02	.005*
*B. Categorical variables*				
Primigravida	18 (41.9%)	16 (39.0%)	20 (44.4%)	.83
Multigravida	25 (58.1%)	25 (61.0%)	25 (55.6%)	.73
Primiparous	29 (67.4%)	22 (53.7%)	28 (62.2%)	.42
Multiparous	14 (32.6%)	19 (46.3%)	17 (37.8%)	.21
RDS	33 (76.7%)	13 (31.7%)	14 (31.1%)	.018*
BPD	19 (44.2%)	3 (7.3%)	5 (11.1%)	.029*
Neonatal death	5 (6.8%)	0 (0.0%)	0 (0.0%)	.14

Presented as mean ± SD or median (IQR); analyzed using ANOVA or Kruskal–Wallis test.

Presented as n (%); analyzed using chi-square or Fisher exact test.

Data are expressed as mean ± SD or median (IQR) for continuous variables and as n (%) for categorical variables.

Statistical comparisons were made using one-way ANOVA, Kruskal–Wallis, or chi-square test as appropriate.

CVAF = cervicovaginal amniotic fluid, PPROM = preterm premature rupture of membranes.

* Statistically significant at *P* < .05.

In contrast, gestational age at delivery and neonatal birth weight showed significant differences between groups. Group 1 exhibited the earliest gestational age at birth (median: 30 weeks [IQR: 26–32]), followed by Group 2 (median: 32 weeks [IQR: 30–34]) and the control group (median: 38 weeks [IQR: 37–39]; *P* < .001). Similarly, mean birth weight was significantly lower in Group 1 (1570 ± 567.6 g) and Group 2 (1892.3 ± 138.2 g) compared to controls (3035 ± 320 g; *P* = .05).

No significant differences were observed in maternal serum concentrations of PDX and nephrin among the groups (*P* = .16 and *P* = .74, respectively). However, CVAF levels of both biomarkers were significantly elevated in the PPROM groups compared to controls. Specifically, CVAF PDX levels were highest in Group 1 (35.05 ± 5.55 ng/mL), followed by Group 2 (23.42 ± 3.77 ng/mL) and controls (10.57 ± 3.21 ng/mL; *P* = .002). Similarly, CVAF nephrin levels were markedly higher in Group 1 (12.88 ± 3.85 ng/mL) and Group 2 (7.58 ± 2.91 ng/mL) than in controls (5.84 ± 2.02 ng/mL; *P* = .005).

Regarding neonatal outcomes, the incidence of RDS was significantly higher in Group 1 (76.7%) compared to Group 2 (31.7%) and controls (31.1%; *P* = .02). BPD was also more frequently observed in Group 1 (44.2%) relative to Group 2 (7.3%) and controls (11.1%; *P* = .03). Neonatal death occurred only in Group 1 (6.8%), but this difference was not statistically significant (*P* = .14).

CVAF PDX showed excellent discriminative ability for identifying classic PPROM cases, with an area under the curve (AUC) of 0.92 (95% confidence interval [CI]: 0.88–0.96), a sensitivity of 93.5%, and a specificity of 92.8% at an optimal cutoff value of 25.17 ng/mL. The diagnostic performance for high PPROM versus control was also strong, with an AUC of 0.82 (95% CI: 0.76–0.88), sensitivity of 85.4%, and specificity of 91.1% at a cutoff of 19.26 ng/mL. The comparison between classic and high PPROM yielded a moderate AUC of 0.79 (95% CI: 0.72–0.86), with 79.1% sensitivity, 89.0% specificity at a cutoff 26.43 ng/mL (Table 2).

**Table 2:** ROC analysis results for CVAF podocalyxin and nephrin.

Comparison	Biomarker	AUC (95% CI)	Cutoff (ng/mL)	Sensitivity (%)	Specificity (%)
Classic vs Control	CVAF Podocalyxin	.92 (.88–.96)	25.17	93.5	92.8
High vs Control	CVAF Podocalyxin	.82 (.76–.88)	19.26	85.4	91.1
Classic vs High	CVAF Podocalyxin	.79 (.72–.86)	26.43	79.1	89.0
Classic vs Control	CVAF Nephrin	.93 (.89–.97)	9.34	96.0	88.9
High vs Control	CVAF Nephrin	.72 (.68–.77)	5.94	79.4	85.9
Classic vs High	CVAF Nephrin	.80 (.74–.86)	8.12	78.8	85.2

AUC = area under the curve, CI = confidence interval, CVAF = cervicovaginal amniotic fluid, ROC = receiver operating characteristic.

Similarly, CVAF nephrin demonstrated excellent diagnostic accuracy for classic PPROM, with an AUC of 0.93 (95% CI: 0.89–0.97), sensitivity of 96.0%, and specificity of 88.9% at a cutoff value of 9.34 ng/mL. For high PPROM versus control, the AUC was 0.72 (95% CI: 0.68–0.77), with 79.4% sensitivity, 85.9% specificity at a cutoff 5.94 ng/mL. In the comparison between classic and high PPROM, the AUC was 0.80 (95% CI: 0.74–0.86), with 78.8% sensitivity, 85.2% specificity at a cutoff 8.12 ng/mL (Table 2).

These results confirm that CVAF concentrations of PDX and nephrin – particularly in classic PPROM cases – possess high diagnostic value and can reliably distinguish between PPROM subtypes and healthy pregnancies.

ROC analyses were performed for each pairwise group comparison using CVAF levels of PDX and nephrin. AUC values with 95% CI, optimal cutoff values, sensitivity, and specificity were calculated using the Youden Index.

**Figure 2.**
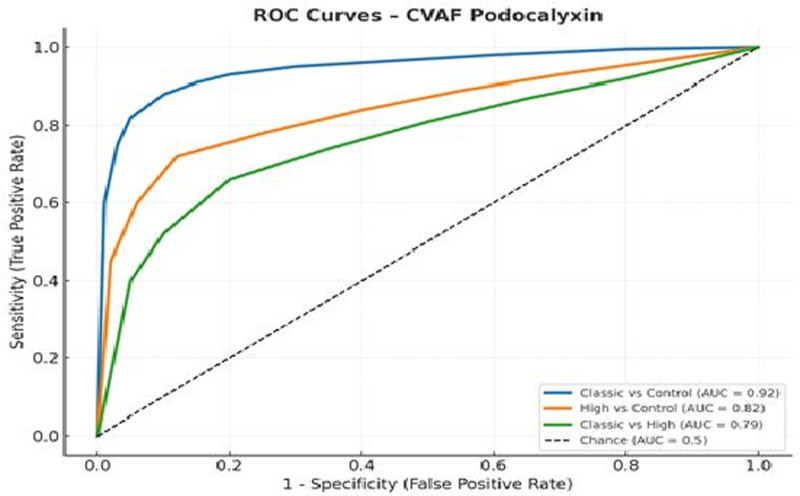
ROC curves for CVAF podocalyxin levels across 3 group comparisons. CVAF = cervicovaginal amniotic fluid, ROC = receiver operating characteristic.

**Figure 3.**
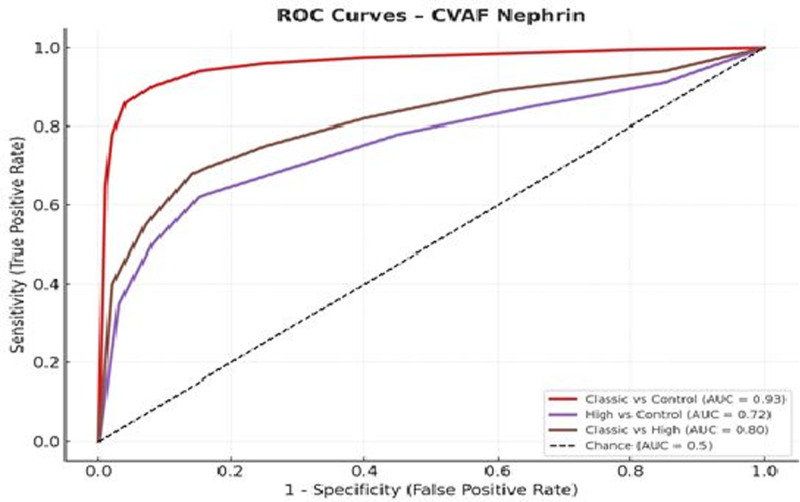
ROC curves for CVAF nephrin levels 3 group comparisons. CVAF = cervicovaginal amniotic fluid, ROC = receiver operating characteristic.

ROC curve analyses demonstrated that both CVAF PDX and nephrin levels demonstrated strong diagnostic performance in differentiating PPROM subtypes from healthy controls (Figs. 2 and 3).

Multivariable logistic regression analysis was performed to identify independent predictors of RDS. Among the tested variables, gestational age emerged as the strongest protective factor. Each additional week of gestation was associated with a 60% reduction in the odds of developing RDS (odds ratio [OR] = 0.40; 95% CI: 0.25–0.65; *P* = .04; Table 3).

**Table 3:** Multivariable logistic regression analysis of factors associated with RDS.

Variable	Outcome	Coefficient (B)	Standard error	*P* value	Odds ratio (exp(B))	95% CI for B	95% CI for OR
Gestational age (wk)	RDS	−0.92	0.23	.04*	0.40	−1.38 to −0.47	0.25 to 0.65
CVAF podocalyxin (ng/mL)	RDS	0.37	0.10	.01*	1.45	0.17 to 0.57	1.18 to 1.77
CVAF nephrin (ng/mL)	RDS	0.17	0.08	.02*	1.18	0.02 to 0.32	1.02 to 1.36
Birth weight (g)	RDS	−0.01	0.01	.04*	0.99	−0.02 to 0.00	0.99 to 1.00
Maternal serum podocalyxin (ng/mL)	RDS	−0.05	0.48	.89	0.95	−0.89 to 0.79	0.47 to 1.92
Maternal serum nephrin (ng/mL)	RDS	−0.09	0.52	.85	0.91	−1.11 to 0.93	0.34 to 2.45

CI = confidence interval, CVAF = cervicovaginal amniotic fluid, OR = odds ratio, RDS = respiratory distress syndrome, ROC = receiver operating characteristic.

* Statistically significant values (*P* < .05).

Elevated CVAF PDX levels were significantly associated with increased RDS risk (OR = 1.45; 95% CI: 1.18–1.77; *P* = .01). Similarly, CVAF nephrin levels were independently predictive of RDS (OR = 1.18; 95% CI: 1.02–1.36; *P* = .02). These findings suggest that local CVAF biomarkers may reflect membrane damage severity and contribute to neonatal pulmonary complications.

Birth weight was also found to be a statistically significant, albeit modest, protective factor (OR = 0.99; 95% CI: 0.99–1.00; *P* = .04), indicating that higher birth weight confers a lower risk of RDS.

In contrast, maternal serum levels of PDX (*P* = .89) and nephrin (*P* = .85) were not significantly associated with RDS risk, and their wide CIs indicate limited predictive utility in this context.

Multivariable logistic regression analysis was conducted to identify independent predictors of BPD. As in the RDS model, gestational age was the strongest protective factor. Each additional week of gestation was associated with a 70% reduction in the odds of BPD (OR = 0.30; 95% CI: 0.18–0.52; *P* = .01; Table 4).

**Table 4:** Multivariable logistic regression analysis of factors associated with BPD.

Variable	Outcome	Coefficient (B)	Standard error	*P* value	Odds ratio (exp(B))	95% CI for B	95% CI for OR
Gestational age (wk)	BPD	−1.20	0.26	.01*	0.30	−1.72 to −0.68	0.18 to 0.52
CVAF podocalyxin (ng/mL)	BPD	0.28	0.09	.02*	1.32	0.10 to 0.46	1.10 to 1.59
CVAF nephrin (ng/mL)	BPD	0.10	0.07	.18	1.10	−0.05 to 0.25	0.95 to 1.28
Birth weight (g)	BPD	−0.01	0.01	.05*	0.99	−0.02 to 0.00	0.99 to 1.00
Maternal serum podocalyxin (ng/mL)	BPD	−0.05	0.48	.89	0.95	−0.89 to 0.79	0.47 to 1.92
Maternal serum nephrin (ng/mL)	BPD	−0.09	0.52	.85	0.91	−1.11 to 0.93	0.34 to 2.45

BPD = bronchopulmonary dysplasia, CI = confidence interval, CVAF = cervicovaginal amniotic fluid, OR = odds ratio.

* Statistically significant values (*P* < .05).

Among the evaluated biomarkers, elevated CVAF PDX levels were significantly associated with an increased risk of BPD (OR = 1.32; 95% CI: 1.10–1.59; *P* = .02). However, CVAF nephrin did not reach statistical significance in the BPD model (OR = 1.10; 95% CI: 0.95–1.28; *P* = .18), suggesting a limited prognostic role in this context.

Birth weight demonstrated a borderline yet significant protective effect (OR = 0.99; 95% CI: 0.99–1.00; *P* = .05), aligning with its known inverse relationship with BPD risk in preterm infants.

Maternal serum levels of PDX and nephrin were not significantly associated with BPD development (*P* = .89 and *P* = .85, respectively), with wide CIs indicating minimal predictive value.

Collectively, the findings highlight gestational maturity and CVAF biomarkers, most notably PDX, as key predictors of BPD in pregnancies complicated by PPROM.

Multivariable logistic regression analysis was conducted to identify independent predictors of BPD. As in the RDS model, gestational age was the strongest protective factor. Each additional week of gestation was associated with a 70% reduction in the odds of BPD (OR = 0.30; 95% CI: 0.18–0.52; *P* = .01; Table 4).

Among the evaluated biomarkers, elevated CVAF PDX levels were significantly associated with an increased risk of BPD (OR = 1.32; 95% CI: 1.10–1.59; *P* = .02). However, CVAF nephrin did not reach statistical significance in the BPD model (OR = 1.10; 95% CI: 0.95–1.28; *P* = .18), suggesting a limited prognostic role in this context.

Birth weight demonstrated a borderline yet significant protective effect (OR = 0.99; 95% CI: 0.99–1.00; *P* = .05), aligning with its known inverse relationship with BPD risk in preterm infants.

Maternal serum levels of PDX and nephrin were not significantly associated with BPD development (*P* = .89 and *P* = .85, respectively), with wide CIs indicating minimal predictive value.

Collectively, the findings highlight gestational maturity and CVAF biomarkers, most notably PDX, as key predictors of BPD in pregnancies complicated by PPROM.

## 4. Discussion

PPROM remains one of the leading causes of perinatal morbidity and mortality, and its diagnosis can be clinically challenging at times.^[15]^ Current diagnostic methods primarily rely on the presence of fluid leakage, which significantly reduces diagnostic accuracy in cases with subclinical or minimal leakage.^[15]^ Therefore, the diagnostic performance of existing techniques is increasingly being questioned, highlighting the need for novel biomarkers that directly reflect the structural integrity of fetal membranes.

Traditional diagnostic methods such as the nitrazine and ferning tests are indirect and often lead to false-positive or false-negative results due to contamination, the presence of blood, or cervical mucus.^[18]^ In contrast, PDX and nephrin are structural biomarkers that directly reflect the biological integrity of fetal membranes.^[4]^ Rather than simply detecting amniotic fluid leakage, these proteins offer a mechanism-based diagnostic approach, indicating local epithelial or endothelial damage.^[10]^ Moreover, unlike conventional biochemical tests that typically detect rupture at more advanced stages, these biomarkers may enable earlier diagnosis in subclinical or high PPROM cases.^[19]^ Notably, even in patients with minimal cervicovaginal leakage, the elevated levels of these proteins suggest not only mechanical rupture but also the presence of sterile inflammation or early microstructural degradation.^[20]^In this context, PDX and nephrin emerge as valuable diagnostic tools, offering higher specificity and potential prognostic value in ambiguous clinical presentations. They may complement or even surpass existing diagnostic tests.

In this research, we aimed to evaluate the diagnostic utility of CVAF levels of PDX and nephrin, transmembrane proteins thought to reflect structural disruption of fetal membranes. Although these proteins have been previously described as markers of renal injury, clinical data on their use in the diagnosis of PPROM are limited.

PDX is a sialomucin glycoprotein belonging to the CD34 family, involved in cellular adhesion, polarity, and epithelial barrier integrity.^[19,20]^ Nephrin is a key component of the slit diaphragm of the glomerular filtration barrier and contributes to cellular cohesion. While PDX is primarily expressed in the kidneys, it has also been detected in the liver and endometrium; nephrin, in addition to renal glomeruli, has been observed in some fetal tissues, including the placenta.^[21,22]^ Histomorphologic and barrier-function similarities have been identified between fetal membranes and the glomerular basement membrane^[22]^ Although studies directly demonstrating the presence of PDX and nephrin in the chorioamniotic membrane are limited, experimental findings suggest their expression in fetal tissues. Notably, nephrin has been shown to be expressed in rat placentas and fetal membranes, and PDX-like proteins have been isolated from human amniotic epithelial cell cultures. Liu et al demonstrated positive expression of CD44, E-cadherin, and PODXL in human amniotic epithelial cells via flow cytometry and immunofluorescence staining.^[23]^ The theoretical relevance of these proteins in the basement layer of the amniotic epithelium supports their potential diagnostic utility.

As these proteins are released into urine upon injury to the glomerular basement membrane, we hypothesized that a comparable disruption in fetal membranes might facilitate their diffusion into the amniotic fluid or cervicovaginal tract. Indeed, while no significant differences were observed in serum levels of PDX and nephrin among groups, their CVAF concentrations showed marked diagnostic utility in differentiating PPROM cases from healthy pregnancies.

Previous studies have shown that biomarker levels in local samples such as cervicovaginal or amniotic fluid tend to increase earlier and more markedly than in systemic circulation. In light of these findings, CVAF levels of PDX and nephrin, like other local biomarkers, may offer superior diagnostic value.^[14,24]^

The ROC analysis demonstrated that CVAF PDX and nephrin levels showed robust discriminatory capacity in all group comparisons, even in clinically challenging high PPROM cases.

Compared to the classic PPROM group, the relatively lower CVAF levels of PDX and nephrin observed in the high PPROM group suggest a more localized and limited pattern of fetal membrane injury. While classic PPROM is characterized by overt membrane rupture and active leakage of amniotic fluid, high PPROM is more often defined by subclinical disruptions in membrane integrity. This clinical distinction may explain why the elevation of CVAF biomarker levels is less prominent in high PPROM cases.

Moreover, the absence of significant differences in maternal serum levels of these biomarkers supports the hypothesis that the pathological process is confined to the fetal membranes and their immediate environment, rather than representing a systemic inflammatory response. This finding underscores the superior diagnostic value of local (CVAF-based) sampling compared to systemic biomarker analyses in conditions involving localized tissue damage, such as PPROM.

These regression-based findings validate and expand upon our earlier hypotheses. While previous studies have suggested that PDX and nephrin play a role in fetal membrane integrity and may have diagnostic and prognostic value in PPROM, our multivariable analyses confirmed CVAF PDX as an independent predictor of both RDS and BPD. This aligns with prior research showing the pro-inflammatory and barrier-disruptive effects of PDX at the endothelial surface.^[25]^

Additionally, we observed a negative correlation between CVAF PDX levels and neonatal birth weight, indicating a potential link between elevated biomarker levels and fetal growth impairment. Similar associations have been reported in conditions such as preeclampsia and intrauterine growth restriction, where endothelial dysfunction contributes to adverse fetal outcomes.^[26,27]^

Although nephrin demonstrated strong diagnostic performance, its prognostic significance appeared to be more limited. In our regression models, nephrin was independently associated with RDS but not with BPD, suggesting it may reflect acute rather than chronic membrane dysfunction.

Importantly, maternal serum concentrations of PDX and nephrin were not significantly associated with either RDS or BPD, and their wide CIs further indicated limited predictive utility. This finding reinforces the superiority of localized CVAF sampling over systemic measurement for evaluating fetal membrane disruption.^[28]^

One of the key design considerations in our study was the possibility that the presence of these proteins in the amniotic fluid might be due to rare fetal glomerular pathologies (e.g., Finnish-type congenital nephrotic syndrome).^[29]^ However, these conditions are rare and are typically identified via antenatal ultrasound findings such as hydrops, intrauterine growth restriction, or oligohydramnios. To exclude this possibility, all neonates in our study underwent postnatal urine and blood biochemical testing, and no evidence of glomerular pathology was observed.

To minimize maternal contamination, all samples were collected using sterile speculum prior to any digital examination, and specimens contaminated with blood, urine, or mucus were excluded from analysis.^[30–33]^

## 5. Conclusion and recommendation

This study highlights the clinical value of CVAF PDX and nephrin levels as noninvasive biomarkers for the diagnosis and risk stratification of pregnancies complicated by PPROM. Both biomarkers demonstrated notable diagnostic performance, particularly in differentiating classic and high PPROM from healthy pregnancies. Moreover, elevated CVAF PDX levels were independently associated with adverse neonatal outcomes such as RDS and BPD, underscoring its potential as a prognostic indicator. Taken together, our findings suggest that PDX and nephrin may serve as *complementary diagnostic tools* to current methods, especially in challenging cases such as high PPROM, with the possibility of broader clinical adoption if validated in larger multicenter studies.

## 6. Strengths of the research

This study has several methodological and clinical strengths that enhance the reliability and applicability of its findings. First, the prospective observational design allowed for real-time data collection, thereby minimizing recall bias and providing insight into the natural course of PPROM under standard clinical management.

Second, the inclusion of both classic and high PPROM subtypes enabled the assessment of diagnostic and prognostic biomarkers within a clinically meaningful classification, which is often overlooked in existing literature. This stratification added a unique dimension to the study.

Third, the analysis of both CVAF and serum samples allowed for a comparative evaluation of biomarker expression at local and systemic levels, offering valuable insights into the pathophysiology of fetal membrane disruption.

In addition, the application of comprehensive exclusion criteria and adherence to standardized sampling protocols improved data validity and minimized potential confounding factors.

From a statistical standpoint, the use of advanced methods such as multivariable logistic regression and ROC curve analysis strengthened the analytical rigor and enhanced the clinical interpretability of the results. Lastly, the investigation of PDX and nephrin – biomarkers that have been underexplored in the context of PPROM – provides an innovative perspective that may contribute to improved diagnostic approaches in obstetric care.

## 7. Limitations

Although this study presents important findings, several limitations should be acknowledged. First, it was conducted at a single center, and the study population consisted exclusively of pregnant women admitted to one hospital. This may limit the generalizability of the findings to broader populations. Another limitation of the study is the relatively small sample size in the high PPROM subgroup (n = 32), which may restrict the generalizability of subgroup-specific findings. Therefore, replication of these results in larger, multicenter cohorts is warranted. Larger, multicenter studies are needed to more robustly assess the diagnostic and prognostic value of these biomarkers. Third, due to the observational design of the study, it was not possible to establish causal relationships; only associations between biomarker levels and neonatal outcomes could be evaluated. In addition, although multiple comparisons were performed, no formal correction for multiplicity (e.g., Bonferroni adjustment) was applied. Since the analyses were hypothesis-driven and focused on predefined outcomes (RDS, BPD, and PPROM subgroup differentiation), the results remain valid; however, the lack of adjustment should be acknowledged as a limitation that may increase the risk of type I error. Finally, the cutoff values used in the analyses were derived solely from this study’s sample and have not yet been externally validated. Therefore, before clinical implementation, these thresholds should be tested in larger and independent cohorts.

## Acknowledgments

The authors would like to thank all patients who participated in the study and the clinical staff at Private Gözde Hospital for their valuable assistance during data collection and sample processing.

## Author contributions

**Conceptualization:** Pervin Karli.

**Data curation:** Pervin Karli.

**Formal analysis:** Pervin Karli, Ümran Karabulut.

**Funding acquisition:** Fatma Tanilir Çağiran.

**Investigation:** Fatma Tanilir Çağiran.

**Methodology:** Ümran Karabulut, Fatma Tanilir Çağiran, Pinar Kirici.

**Project administration:** Ümran Karabulut, Fatma Tanilir Çağiran, Pinar Kirici, Serhat Ege.

**Resources:** Pinar Kirici, Serhat Ege.

**Software:** Serhat Ege.

**Supervision:** Serhat Ege.

**Validation:** Zercan Kali, Serhat Ege.

**Visualization:** Zercan Kali, Pervin Karli.

**Writing – original draft:** Zercan Kali, Pervin Karli.

**Writing – review & editing:** Zercan Kali, Pervin Karli.
